# Administrative Process and Criteria Ranking for Drug Entering Health Insurance List in Iran-TOPSIS-Based Consensus Model

**Published:** 2016

**Authors:** Amir Viyanchi, Ali Rajabzadeh Ghatari, Hamid Reza Rasekh, HamidReza SafiKhani

**Affiliations:** a *School of Pharmacy, Hamedan University of Medical Sciences, Hamedan, Iran. *; b *Tarbiat Modares University, Tehran, Iran. *; c *School of Pharmacy, Shahid Beheshti University of Medical Sciences, Tehran, Iran.*; d *Strategic Council at National Research Network for Policy Making, Economics & Health Management, Tehran, Iran.*

**Keywords:** Health Insurance, Criteria Reimbursement, Decision Making, Iran

## Abstract

The purposes of our study were to identify a drug entry process, collect, and prioritize criteria for selecting drugs for the list of basic health insurance commitments to prepare an “evidence based reimbursement eligibility plan” in Iran.

The 128 noticeable criteria were found when studying the health insurance systems of developed countries. Four parts (involving criteria) formed the first questionnaire: evaluation of evidences quality, clinical evaluation, economic evaluation, and managerial appraisal. The 85 experts (purposed sampling) were asked to mark the importance of each criterion from 1 to 100 as 1 representing the least and 100 the most important criterion and 45 out of them replied completely. Then, in the next questionnaire, we evaluated the 48 remainder criteria by the same45 participants under four sub-criteria (Cost calculation simplicity, Interpretability, Precision, and Updating capability of a criterion). After collecting the replies, the remainder criteria were ranked by TOPSIS method. Softwares “SPSS” 17 and Excel 2007 were used.

The ranks of the five most important criteria which were found for drug approval based on TOPSIS are as follows: 1-domestic production (0.556), 2-duration of using (0.399), 3-independence of the assessment group (0.363) 4-impact budgeting (0.362) 5-decisions of other countries about the same drug (0.358). The numbers in parenthesis are relative closeness alternatives in relation to the ideal solution. This model gave a scientific model for judging fairly on the acceptance of novelty medicines.

## Introduction

Iran’s population is 75.1 million (1). Pharmaceutical market size is 4200 million United State Dollar (USD) and spending on pharmaceuticals per capita is 48 USD. The share of pharmaceutical costs as a part of Gross Domestic Production (GDP) was 1.1 % in 2010. Iran’s GDP per capita was 7,310USD in 2012 (1), also the mean life expectancy for both male and female persons in the same year, was 71 years (2). Financing the secondary and tertiary-level curative care, are sometimes provided directly through the compulsory Social Security Organization for formal sector employees and their dependants, the Armed Forces Medical Services Organization for the militaries and their families, and the Iranian health insurance organization for government employees, rural households, the self-employed, and Others (for example students) (3).

Pharmaceutical costs and related expenses in Iran and several developing countries, is about 30 percent of total health care spending and comprises nearly 50 percent of the cost of outpatient care(4). The share of insurances in public health related payments in Iran is only 18% and according to the statements of the previous health minister, health insurers must accept the cost of expensive medications (5). Today, in regard to the developments in the health insurance industry and increase in the number of people that are covered by them in the country (table1), although it promotes participation in financing but on the other hand it also raises the costs of the industry. So to balance the income and expenses it is required that the acceptance of new service obligations by insurance organizations including medication services be logical, scientific, and transparent.

**Table 1:** Population covered by categorization of Iran's insurance funds

**Row**	**Fund name**	**Persons covered**
1	Social security organization	27.830.916
2	Iranian health insurance organization)prior MSIO)	36.500.000
3	Organization of health services of the armed forces	4.500.000
4	Imam Khomeini's Relief Committee (RA)	2.000.000
The sum total of: 70.830.916 People		

Adopted:        Ibrahimipour H. 2011 )

**Figure 1:**
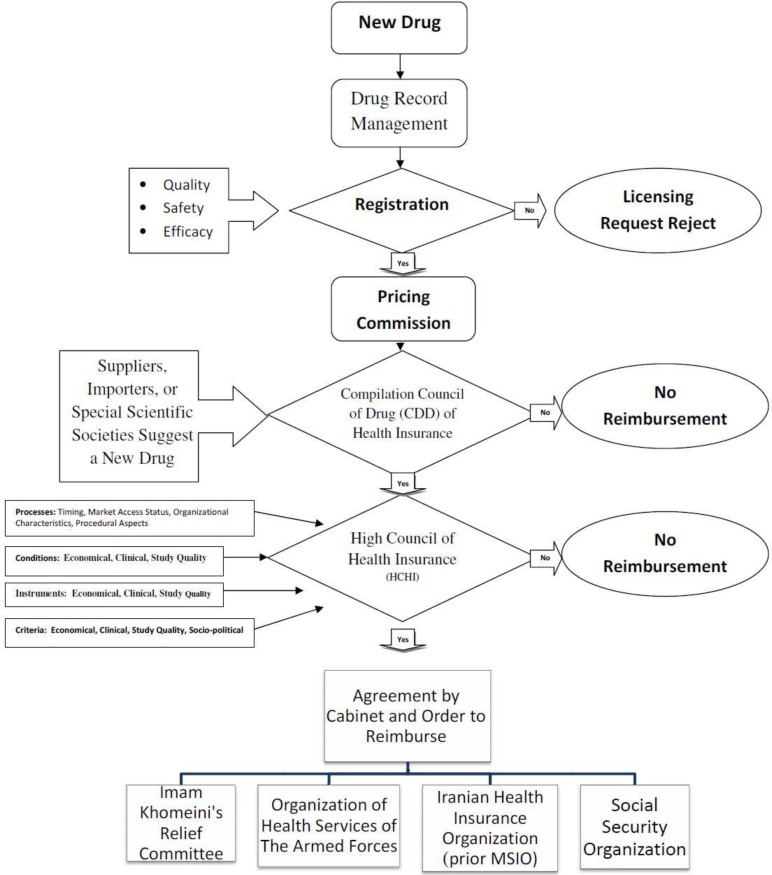
New drug registration process in national pharmaceutical list and reimbursement process in basic health insurance in Iran.

However, medications in Iran before obtaining the consumption license must be registered in the registration list of medicines by the Drug Selecting Committee of the drug deputy of Ministry of Health (MOH). After registration of the new drug in the list of authorized medicines, suppliers, importers, or special scientific societies in the medical field can suggest a new drug to be put into the coverage list of insurers (Figure 1). Applicants have to submit their request to “Compilation Council of Drug” (CCD). As process of evaluating, requests along with their documentations are expressed in the CCD of insurance organizations. Members of this council consist of permanent members like insurance organizations delegates, ministry of welfare and social security delegates, and vice president for strategic monitoring delegate. There are non-permanent members without the right to vote, with an advisory role such as scientific institutions representatives, the medical council, and if needed the pharmacopeia council members of the health ministry will be invited by the CCD. 

The CCD's tasks are to scrutinize the list of drugs covered by insurers in order to preserve the insured right of access to essential drugs, in accordance with the financial resources of insurance organizations, prioritizing the health needs of the insured and enhancing public health services performance in prevention of forcing intolerable costs to the insured. CCD recommendations are according to a majority vote by a multidisciplinary expert committee and are based on evaluation of pharmaco-economic studies submitted by manufacturers or other eligible applicants seeking reimbursement. CCD’s assessments should be evidence-based. The guidelines published by the Iranian Dept. of Health Technology Assessment and MOH guidelines for economic evaluation of pharmaceuticals are recommended for preparing submissions for the CCD (6). If a consensus is achieved in the CCD, the proposal along with its documentation will be sent to the High Council of Health Insurance (HCHI) of the country's medical services insurance. They will make a decision about whether the drug will be added to the list of the medical insurance organizations and whether it is allowed for prescription by general physicians or special experts are only permitted to do so and should it be used in particular clinics or not?

The HCHI will get the approved list confirmed by the cabinet for implementation, then will notify four general insurance organizations. HCHI also prepares an official list of drugs called the negative list (drugs that don’t contain the possibility of being covered by insurances in Iran) (7).

Unfortunately, in Iran except registration forms, things like the time frame, regulations, and conditions of reviewing the applications are not clear and decisions are mostly based on individuals' opinions instead of scientific evidence. We tried to find standards and criteria that were noticeable in health insurance organizations of developed countries. A criterion is a distinguishing quality that determines the value of a technology. For instance, some criteria are clinical effectiveness, the impact of budgets, cost effectiveness, or severity of the disease and... (table 2). Besides the definition of a criterion, decision-makers must also specify the value of those criteria, and before being able to judge about a certain technology, they must know what is “good enough” (8). Just like cost effectiveness, a criterion may be expressed with a threshold value**.**

**Table 2:** Key criteria used by some countries to make drug coverage decisions, 2008

**Decision criteria**	**Denmark**	**England**	**France**	**Germany**	**Netherlands**	**Sweden**
Therapeutic benefit	●	●	●	●	●	●
Cost effectiveness		●		●	●	●
Necessity (disease burden, severity)				●		●
Availability of treatment alternatives		●		●	●	●
Public health impact			●			
Equity		●				●
Innovative characteristics (for example, the ease of use)		●	●		●	
Budget impact		●	●		●	
Ethical/legal considerations		●			●	
Feasibility of assessment		●				

(Adopted: Corinna Sorenson 2010)

* It is not clear

## Experimental

*Materials and methods*

In this study, the criteria and standards for admission of medications in the coverage list of insurance organizations in developed countries were extracted and collected in four main categories by studying the articles published in credible world journals or official associated websites or by opinion leaders. We evaluated and prioritized criteria based on the opinions of former and current members of CCD and members of the HCHI and also the elites of the health care system using the Technique for Order Preference by Similarity to an Ideal Solution (TOPSIS) method, to make a model to help decision makers for preferring new drugs for Iran’s health insurances. Modeling is a theoretical framework for analysis of acts of decision-making and it represents the rational decision-making in the health care. Economic models provide a tool that with them the foundation of decisions can be placed in front of everyone's sight. In brief, the economic models create a logical framework in which the best available evidences to pass on decisions about selection and reimbursement of medicinal products can be used. Probable approaches for evaluating the uncertainty around the activity and the variables in the models increase the usefulness of the results for decision makers (9). The four categories that were used to divide the studied literature were quality evaluation, clinical evaluation, economic evaluation, and managerial appraisal. In the first stage, a questionnaire was constructed based on 128 extracted criteria and participants were asked to mark the importance of each criterion from 1 to 100. A simple weight elicitation technique on a 100-point scale was selected for this investigation, with 1 representing the least and 100 the most important criterion. The questionnaire was measured in terms of validity and reliability before distribution and its score validity was 96% in cronbach scale. The questionnaires were distributed directly, or via the Internet between 85 individuals (purposed sampling) of current and previous members of CCD and HCHI and some elites of health system management. Forty-five individuals completed the questionnaire (table 3). Any criteria that had a mean less than 60 and a standard deviation more than 25 were excluded at this stage and another questionnaire was prepared with the 48 remainder items (table 4) using 4 "fixed sub-criteria" for each criterion as the second stage. The 4 fixed sub- criteria are defined as below:

1- The feasibility of determining the cost of obtaining a criterion: How easy it is to determine the cost of a criterion?

2- Criterion Interpretability: How easy it is to interpret if the value of a criterion is high or low and would it be simple to understand? Interpretation of some of the criteria is difficult because either relatively high or low values of the criteria may imply poor performance (e.g., the proportion of recurrent costs absorbed by drugs).

3- Criterion Precision: How much is the criterion clearly defined to implicate all aspects of the subject?

4- Criterion timeliness: to what extent the criterion can regularly, periodically, and also immediately be prepared? 

This questionnaire was sent to the same participants (n = 45) after going under the validity and reliability tests, and answers were obtained on a 5 point LIKERT scale (1 – very little 2 –little 3 – medium 4 – much 5 – very much). After collecting replies, the main criteria were ranked by TOPSIS, which is one of the multi-criteria decision-making methods and so the ranking of the criteria was determined.

**Table 3:** Demographic specifications of responders to questionnaires

**variable**	**range**	**n**	
Gender	Male Female	28 17	
Age	<40 =>40, <=45 >46, <=50 >50	15 18 8 4	
Education	Master MD PhD	3 26 16	
Cross study	Physician Pharmacy Para-medical sciences	7 31 7	
Work experiment(year)	O <3 >=3,<=10 >=11,<=15 >=16,<=20 >20	2 4 7 9 12 11	
experiment (year) in CCD	O <3 >=3,<=10 >=11,<=15	27 7 9 2
experiment(year) In HCHI	O <3 >=3,<=10 >=11,<=15	28 8 7 2

**Table 4:** Important criteria list which formed items of second questionnaire

**criterion**	**description**
A1	The existence of the pharmacoeconomy assessment
A2	Announcement of the incremental cost effectiveness (ICER) of a new drug
A3	Impact budget
A4	Reasonableness of increasing insurance costs due to acceptance of new drug
A5	Being a reasonable price of a new drug
A6	Sales volume of the new drug in society covered by the insurance not be more than the current volume
A7	domestic production of a new drug
A8	The existence of physician demand
A9	Declining the cost of medical services (by reducing the use of other hospital services, Para-clinical and ...)
A10	Make the most of living healthier
A11	Threshold setting About the new drug cost effectiveness. (For example, 16 thousand USD for one year of healthy life)
A12	The existence of a proper clinical trial evidence to determine the effectiveness of the new drug
A13	Assurance of the increased survival
A14	Prevent death and further disability
A15	Effectiveness of new drugs
A16	Safety of new drugs
A17	Considerable decreasing the number of patients, recovered by new drug
A18	The existence of comparable medication
A19	Being vital medication
A20	Conducting age-targeting
A21	Specifying target population
A22	The ability to help in the reduction of health hazards by the new drug.
A23	Public health promotion
A24	The new drug benefit For other applications (other indications)
A25	Be effective in reducing other interventions
A26	Much easier to use a new drug
A27	Shorter use duration of the drug in comparison with the similar drug
A28	accomplish cost-effectiveness study for new drugs entering to Insurance List
A29	Independence of Assessment Group
A30	The high quality of the scientific literature reviewed in the study
A31	Determination the validity of the economic model
A32	Assessment of the viewpoint of stakeholders (patients, pharmaceutical companies, insurers, government and society), in the study
A33	Determination of the degree of uncertainty in economic evaluation studies
A34	Consideration of health technology assessment (HTA)
A35	Recently published study of a new drug
A36	The characteristics of drug use in low income deciles
A37	The existence of a history of drug use in similar situation countries to us
A38	Observe the ethical considerations (rule of rescue)
A39	The existence of a positive opinion of manager(s) to accept new drug
A40	Assurance of continuous availability of new drug
A41	The drug is in priorities of disease treatment
A42	The drug is in priorities of disease screening
A43	The drug is in priorities of disease diagnosis
A44	The drug is in priorities of disease prevention
A45	The existence of favorable comments of the expert committee in the process of the admissions decision for medicine
A46	Providing a rational response for decision report to people
A47	Providing a rational response to the revise decision
A48	Having a history and previous drug assessment at the council reviews of the insurer

*The TOPSIS method*

TOPSIS method is presented by Chen and Hwang (10), with reference to Hwang and Yoon (11). TOPSIS is a multiple criteria method to recognize solutions from a limited set of alternatives. The fundamental rule is that the preferred alternative should have the shortest distance from the ideal solution and longest distance from the negative-ideal solution. The method of TOPSIS can be expressed in a sequence of steps: 1) Calculate the normalized decision matrix. 2) Calculate the weighted normalized decision matrix. 3) Determine the positive-ideal and negative-ideal solution. 4) Calculate the separation measures, using the n-elemental Euclidean distance. 5) Calculate the relative closeness to the ideal solution. Its value can extend from zero to one (table5 and 6) Rank the preference sort. For ranking alternatives using this table, we can rank them in descending order (table 6). The TOPSIS method introduces two “reference” points (12).

**Table 5:** The relative proximity quantity of criteria and extracted standards with the TOPSIS model

***A***_i_	**relative proximity**	***A***_i_	**relative proximity**
A1	*0.334*	A25	*0.249*
A2	*0.306*	A26	*0.326*
A3	*0.362*	A27	*0.399*
A4	*0.305*	A28	*0.302*
A5	*0.318*	A29	*0.363*
A6	*0.352*	A30	*0.251*
A7	*0.556*	A31	*0.227*
A8	*0.323*	A32	*0.208*
A9	*0.236*	A33	*0.231*
A10	*0.322*	A34	*0.281*
A11	*0.279*	A35	*0.223*
A12	*0.308*	A36	*0.276*
A13	*0.309*	A37	*0.358*
A14	*0.322*	A38	*0.269*
A15	*0.352*	A39	*0.337*
A16	*0.354*	A40	*0.310*
A17	*0.271*	A41	*0.314*
A18	*0.332*	A42	*0.215*
A19	*0.318*	A43	*0.212*
A20	*0.284*	A44	*0.241*
A21	*0.315*	A45	*0.333*
A22	*0.297*	A46	*0.227*
A23	*0.276*	A47	*0.266*
A24	*0.214*	A48	*0.351*

**Table 6:** The final criteria ranking and extracted with TOPSIS model

***Rank***	***Alternatives***	***A +***	***d +***	***d-***	***Rank***	***Alternatives***	***A +***	***d +***	***d-***
*1*	*A7*	*0.556*	*0.034*	*0.042*	*25*	*A2*	*0.306*	*0.048*	*0.021*
*2*	*A27*	*0.399*	*0.042*	*0.028*	*26*	*A4*	*0.305*	*0.045*	*0.020*
*3*	*A29*	*0.363*	*0.045*	*0.026*	*27*	*A28*	*0.302*	*0.047*	*0.020*
*4*	*A3*	*0.362*	*0.045*	*0.025*	*28*	*A22*	*0.297*	*0.047*	*0.019*
*5*	*A37*	*0.358*	*0.045*	*0.025*	*29*	*A20*	*0.284*	*0.047*	*0.019*
*6*	*A16*	*0.354*	*0.045*	*0.025*	*30*	*A34*	*0.281*	*0.047*	*0.019*
*7*	*A15*	*0.352*	*0.045*	*0.025*	*31*	*A11*	*0.279*	*0.049*	*0.019*
*8*	*A48*	*0.351*	*0.045*	*0.024*	*32*	*A36*	*0.276*	*0.048*	*0.018*
*9*	*A6*	*0.352*	*0.045*	*0.023*	*33*	*A23*	*0.276*	*0.048*	*0.018*
*10*	*A39*	*0.337*	*0.045*	*0.023*	*34*	*A17*	*0.271*	*0.049*	*0.018*
*11*	*A1*	*0.334*	*0.047*	*0.023*	*35*	*A38*	*0.269*	*0.047*	*0.017*
*12*	*A45*	*0.333*	*0.045*	*0.023*	*36*	*A47*	*0.266*	*0.047*	*0.018*
*13*	*A18*	*0.332*	*0.046*	*0.023*	*37*	*A30*	*0.251*	*0.048*	*0.016*
*14*	*A26*	*0.326*	*0.046*	*0.022*	*38*	*A25*	*0.249*	*0.049*	*0.016*
*15*	*A8*	*0.323*	*0.044*	*0.021*	*39*	*A44*	*0.241*	*0.051*	*0.016*
*16*	*A10*	*0.322*	*0.047*	*0.022*	*40*	*A9*	*0.236*	*0.050*	*0.016*
*17*	*A14*	*0.322*	*0.046*	*0.022*	*41*	*A33*	*0.231*	*0.050*	*0.015*
*18*	*A19*	*0.318*	*0.046*	*0.021*	*42*	*A31*	*0.227*	*0.050*	*0.015*
*19*	*A5*	*0.318*	*0.047*	*0.022*	*43*	*A46*	*0.227*	*0.050*	*0.015*
*20*	*A21*	*0.315*	*0.046*	*0.021*	*44*	*A35*	*0.223*	*0.050*	*0.014*
*21*	*A41*	*0.314*	*0.046*	*0.021*	*45*	*A42*	*0.215*	*0.052*	*0.014*
*22*	*A40*	*0.310*	*0.047*	*0.021*	*46*	*A24*	*0.214*	*0.050*	*0.014*
*23*	*A13*	*0.309*	*0.047*	*0.021*	*47*	*A43*	*0.212*	*0.052*	*0.014*
*24*	*A12*	*0.308*	*0.046*	*0.021*	*48*	*A32*	*0.208*	*0.050*	*0.013*

## Results

**Figure 2:**
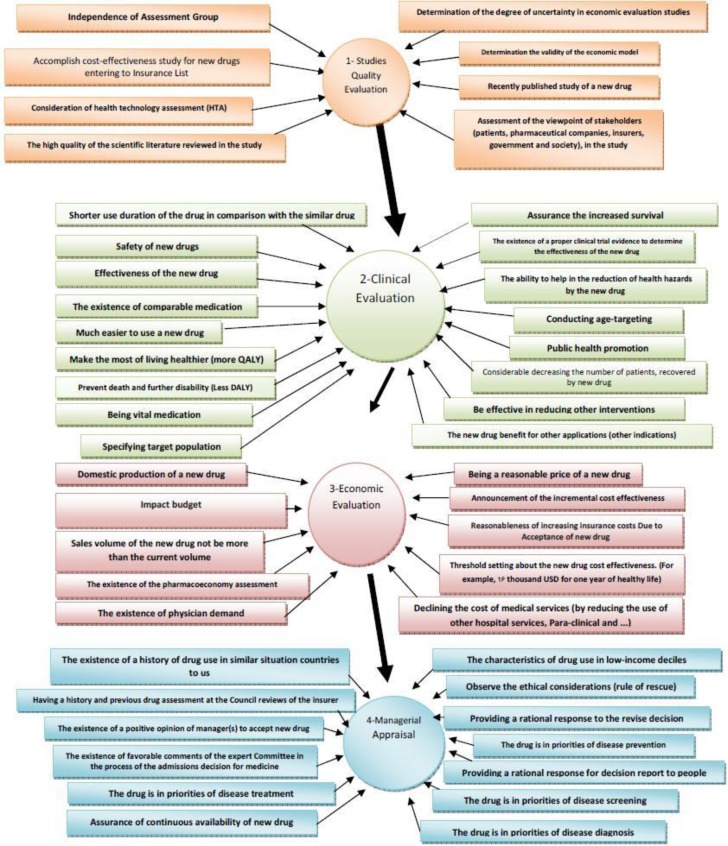
The conceptual model corresponds to the ranking criteria in the second part of the study

In the first stage, results of classifying and ranking the criteria were obtained in 4 sections of: qualitative evaluation of the studies (M =79.64, SD =19.84), clinical evaluation (M =78.96, SD =19.42), managerial appraisal (M = 75.16, SD = 22.58) and finally, economic evaluation (M = 71.63, SD =24.03). The conceptual design of the effects of each of the obtained criteria from these four parts on the process of decision-making over admission of drugs into the list of health insurance obligations, after employing TOPSIS that led to repositioning economic evaluation before managerial appraisal, is shown in figure 2. At this point, the obtained pattern is as follows: documents related to a new drug will be delivered to CCD for performing initial reviews on the quality of the performed studies and the way they are done by the manufacturer or the importer. CCD will send the documents to expert (M=85.27, SD=14.3) and independent committees (M=83.27, SD=19.27) for scientific investigation and these committees will announce their opinion to CCD. There was no consensus over establishing a deadline for these committees to respond (SD=28.6). If the quality of the study is confirmed, then the drug will be sent to the clinical evaluation committee for clinical assessment. After assessing the documents related to clinical trials of the drug and confirming the veracity of the claims related to its clinical effectiveness, the opinions of the committee will be reported to the secretariat of CCD. CCD makes its managerial decision with applying managerial views and opinions such as whether the new medication can be placed into the list of strategies of insurer organizations or not? (M=74.16, SD=22.54); whether the drug has the characteristics of consumption in the lower income deciles or not? (M=73.96, SD=27.46) and so on. If a drug is favorable, then the CCD has to seek for economic results and costs associated with the new drug. These reviews begin by submitting the data to the economy committee of the drug. This committee assesses economical feasibility of admission of the medication and reasonable prices to begin undertaking the new drug in the basic health insurances by using the available criteria and if necessary, while accepting the drug, the committee sends its conditions and suggestions to CCD. At the second stage of the investigation, the obtained criteria were re-ranked by the TOPSIS model with the help of certain sub-criteria that in next we would describe the top 5 criteria.

## Discussion

1- Domestic production of new drugs: This criterion achieved the highest rank in TOPSIS method and its relative closeness to the ideal solution was 0.556, which has the best value among other criteria. It can be said that a domestic new drug will be worth 49-1=48 points. 

The dilemma for health insurance organizations is to find a policy that can find a balance between the objectives of ensuring. This policy is making drugs affordable to the people, and health insurances funds, and to aid to promote and develop domestic drug production without threatening the revenue generation activities of medical providers, while keeping the macro policies of the government firm.

Because, health insurance funds, with their huge outlay on drugs each year (30% out of total budget), were keen to have greater control over drug prices and volume. There was a tendency for drug importers and doctors to introduce foreign drugs to the commitment list of health insurances. But CCD severely restricted it, since a domestic drug production policy is determined by HCHI. There are measures in place to restrict spending on pharmaceuticals such as popularizing prescribing domestic drugs. While, patients and doctors both want greater freedom to get the best drugs available, especially if health insurance is paying for them. 

 The local pharmaceutical industry had tried to develop and gradually increase its market share with an explicit policy goal to reach 84% of market share by 2025 (13). Most of its production consisted of low-priced generic drugs, although the government was providing some assistance for innovation, especially for industrializing the production of traditional herbal medicines. Such criteria for selecting drugs for reimbursement favored domestic producers. 

2- Duration of using the new drug: This criterion owns the second rank according to the sub criteria and the TOPSIS method. The relative proximity number of this criterion is 0.399. If the average duration of using the new drug is shorter than the average duration of using the previous drug (if it existed) then the drug will also have 49-2=47 points at this stage. This criterion is included in the clinical assessment criteria.

Because duration of using a drug has effects on consuming quantity and drug quantity is one of the cost elements, so the shorter the treatment duration by the new drug becomes, it causes lesser costs directly for insurances funds.

On the other hand, short duration of using a drug, can probably create less adverse drug reactions. Adverse drug reactions are a leading cause of morbidity and represent a substantial burden on health-care resources. Many countries spend 15% to 20% of their hospital budgets to treat drug complications (14, 15). Therefore, this criterion is a good factor to indirectly diminish costs for health insurance Companies. 

Also in Canada, in order to assess requests accurately for drug coverage, the following information is required: number of days per cycle for each agent, cycle frequency, and expected treatment duration (total number of cycles) (16).

3-The results of intra-country studies (clinical and economic) for a new drug get evaluated by an independent group: from the perspective of TOPSIS method if a drug gets evaluated by an independent appraisal group and gets suggested for joining the drug commitment list of insurance organizations, it will have the score of 46. Its relative closeness number to the ideal solution was 0.363 and it is close to the positive ideal of “Very much“ more than the remained criteria. This criterion is classified under the category of quality evaluation of a study. 

Like this, Danko suggested for a balanced assessment system in middle-income countries. The pricing and reimbursement body (PRB) performs a scientific inspection and, if the profile is complete, it forwards the entire documentations to a health technology assessment group (HTAG) that, if possible, works independently from the PRB. Between PRB and an HTAG is a major distinction. A PRB is a decision-making body that consults with pharmaceutical companies and takes pricing and reimbursement decisions, whereas an HTAG is a specialist association that carries out a balanced assessment system which is a very important input for the PRB’s following decision. HTAGs are therefore the excellent arrangers and specialized independent organizations, government officials. Either they are under the support of scholastic institutions or they are not. PRB should not affect on HTAG decisions; otherwise, the PRB’s position and last budget-related regards would influence the fairness of the HTAG assessment. Ideally, HTAGs themselves can be comprised of two subgroups, the first one performing simplified economic evaluations, and the second one reviewing the worth of novel drug or new equipment for patients and community (17). Another utilization instance of independent groups is in Canada. Drug Quality and Therapeutics Committee (DQTC) is an expert advisory group to provide independent and specialized advice to the Minister of Health of this country. In 1997, a Sunset Review was conducted of the DQTC to re-evaluate the continued role and mandate of the committee. One of the major recommendations of the review panel was that representation from health economics and pharmaco-epidemiology should be increased to enhance the committee’s focus on the value for money assessment of new drugs (18).

 Therefore, it is logical that we separate assessment groups in specialized categories for new drug entering to reimbursement list of public health insurances and these groups must have no dependence to supply side stakeholders and providers. 

4- Impact budgeting: Economic criteria for drug evaluation entail this criterion. The Budget Impact Analysis is one of the key elements of the reimbursement dossier which was placed among the top rankings, and its relative closeness number (0.362) got the forth place. In brief, the Budget Impact Analysis can be described as an accurate estimate of predicted incremental expenditures following reimbursement of the new drug (19). In the event that the economic committee of the council (CCD) predicts that the costs of a new drug are affordable for the budget of health insurance organizations, this criterion will get 45 points among other criteria.

In Canada, manufacturers appealing reimbursement by public drug plans are necessitated to produce an ample submission about budget impact analysis more over other evidences like the clinical, the burden of illness, the costs associated with the disease, a systematic review of literature, and the cost-effectiveness of the product (19). Benefits forgone are the economic and equity reasoning for performing budget impact analyses. To state the matter differently, by choosing to draw down the budget in one way, decision makers relinquish other opportunities to use the same resources. In addition, if the target is not to maximize health gains subject to a budget or resource control, but to decrease variance in health gains, budget impact analysis is more useful to the decision maker than cost-effectiveness analysis (20). 

 Budget Impact Analysis as an effective, practical financial tool has been introduced to the policy makers for improving drug formulary and reimbursement decision making (21). However, policy makers do not easily admit that they consider budget impact and are even reluctant to explicitly use budget impact as a formal criterion. A debate would strengthen the theoretical foundation of budget impact as a legitimate criterion in the context of drug reimbursement decisions. Such discussion of budget impact's role will also enhance policy-makers' accountability (22).

5-Decision of other countries with social-economic status similar to Iran in relation to a new drug’s reimbursement (international reimbursement imitational): this is the first managerial and social criterion for drug admission that got the fifth rank with relative closeness number of 0.358. If a new drug is accepted by health insurances of those countries that have economic and social situations similar to Iran, this criterion will acquire 44 points and if it is not approved by them, it will be deprived from these points.

Most middle-income countries seem to have chosen their peer countries based on global academic representations (e.g., UK, Canada, Australia-not independently from the influence of English-language HTA literature), cultural links, and know-how transfers via international development initiatives (e.g., France, Sweden or, to a lesser extent, the Netherlands) (23, 24).

Ranking of the remainder of the criteria is shown in table6. Finally, a new drug that could gain 588 points out of the total of 1176 points from different criteria can be sent with a positive consideration from the CCD of insurer organizations to the HCHI. 

*Limitations of the study*

Admittedly, this study may suffer from some limitations. This study is based on elites and experts’ intuitive decision-makings. Direct access to documents and evidences about prior decisions in CCD was confidential or classified. So, the authors could not find drug dossiers that support or reject the results. Results generalization is limited, because research samplings were mostly from Tehran. Finally, to reason of imperative entering data for all items in TOPSIS method, we had to give 1 as a minimum value for a few items that were non-respond. 

## Conclusion

 According to the experts, if the government want pharmaceutical industry to grow in the long run and be effective as a strategy for a prosperous national economy in the twenty-first century, it is necessary that we use scientific criteria for decision making about putting new drugs into the national reimbursement list and insurance companies coverage list. In this paper, we have developed a new method for multiple attribute decision-making in the drug realm with a TOPSIS-based consensus process and so a method has been developed for drug preferring. Although the study is based on the subjective views of participants, the survey results are considerable. Due to the fact that the participants are authoritative persons with experience and high educations, the obtained model can be used as a pilot in a specific period until its actual results get assessed and by using the quantitative data obtained from this experiment, the model can then be reviewed and analyzed. This model has given a scientific framework for judging fairly on the acceptance of novel medicines into the health insurance organization's commitment without relying on the usual trial and error method but based on a certain procedure and a roadmap, with the lowest rate of error. We hope that this study would offer a toolkit and prepare an “evidence based reimbursement eligibility plan” for policy.

## References

[1] Trading Economics, Iran | Economic Indicators.

[2] World Life Expectancy,Country Health Profiles, Health Profile: Iran.

[3] Rahbar M (2006). Series of Health Reports in Islamic Republic of Iran.

[4] Mohsenpour SR, Basmangi K, Izadi S, Behbahani AA (2005). Abstract study of pharmaceutical in IRI. Health commission of the IRI parliament. No report: 7608.

[5] Vahid-dastjerdi M (2011). Interview with IRNA reporter in 12 Oct.

[6] Doaee SH, Olyaeemanesh A, Emami SH, Mobinizadeh M, Abooee P, Nejati M, Zolani GS (2013). Development and implementation of health technology assessment: A policy study. Iran J Pub Health.

[7] Delgoshaee B (2004). Comparison of drug pricing system and drug insurance in Iran and selected countries. J Health Administration.

[8] Giacomini M (2007). How good is good enough? Standards in policy decisions to cover new health technologies. Healthcare Policy.

[9] Bryan S, Sandercock J, Barton P, Burls A, Freemantle N, Hill S (2004). Evaluating pharmaceuticals for health policy and reimbursement. Tensions in Licensing and Reimbursement Decisions: The Case of Riluzole for Amyotrophic Lateral Sclerosis.

[10] Chen SJ, Hwang CL (1992). Fuzzy Multiple Attribute Decision-Making: Methods and Applications.

[11] Hwang CL, Yoon K (1981). Multiple Attribute Decision-Making Methods and Applications.

[12] Jahanshahloo GR, Hosseinzadeh Lotfi F, Izadikhah M (2006). Extension of the TOPSIS method for decision-making problems with fuzzy data. Appl Mathematics Comput.

[13] Kebriaeezadeh A (2012). Pharmaceutical vision of Iran in 2025. Sepid.

[14] Bordet R, Gautier S, le Louet (2001). Analysis of the direct cost of adverse drug reactions in hospitalized patients. Eurff Clin Pharmacol.

[15] White TJ, Arakelian A, Rho J (1999). Counting the costs of drug-related adverse events. Pharmacoeconomics.

[16] (2014). Newfoundland Labrador: Health and Community Services site.

[17] Danko D (2014). Health technology assessment in middle-income countries: recommendations for a balanced assessment system. J Market Access Health Policy.

[18] (2000). Ministry of Health and Long-Term Care (Canada).Ontario Guidelines for Drug Submission and Evaluation.

[19] Hubert MM (2014). Pharmacoeconomics: Five Simple and Effective Ways to Optimize Budget Impact Analyses and Obtain Drug Reimbursement.Xtalks Webinar.

[20] Cohen JP, Stolk E, Niezen M (2008). Role of budget impact in drug reimbursement decisions. J Health Polit Policy Law.

[21] Jamshidi HR, Foroutan N, Salamzadeh J (2014). “Budget Impact Analyses”: A Practical Policy Making Tool for Drug Reimbursement Decisions. Iran J Pharm Res.

[22] Niezen MG, de Bont A, Busschbach JJ, Cohen JP, Stolk EA (2009). Finding legitimacy for the role of budget impact in drug reimbursement decisions. Int J Technol Assess Health Care.

[23] Viyanchi A, Rasekh HR, Safikhani HR, Rajabzadeh A (2015). Drug Insurance Coverage in Iran and Some Selected Countries: A Comparative Study. J Health Adm.

[24] Oortwijn W, Mathijssen J, Banta D (2010). The role of health technology assessment on pharmaceutical reimbursement in selected middle-income countries. Health Policy.

